# Rapid total volatile organic carbon quantification from microbial fermentation using a platinum catalyst and proton transfer reaction-mass spectrometry

**DOI:** 10.1186/s13568-016-0264-2

**Published:** 2016-10-05

**Authors:** Heidi R. Schoen, Brent M. Peyton, W. Berk Knighton

**Affiliations:** 1 Department of Chemical & Biological Engineering, Montana State University, 305 Cobleigh Hall, PO Box 173920, Bozeman, MT 59717 USA; 2 Center for Biofilm Engineering, Montana State University, 366 Barnard Hall, P.O. Box 173980, Bozeman, MT 59717 USA; 3 Department of Chemistry and Biochemistry, Montana State University, 103 Chemistry and Biochemistry Building, PO Box 173400, Bozeman, MT 59717 USA

**Keywords:** Fungal endophyte, Proton transfer reaction-mass spectrometry, Solid state fermentation, Total volatile organic carbon

## Abstract

A novel analytical system was developed to rapidly and accurately quantify total volatile organic compound (VOC) production from microbial reactor systems using a platinum catalyst and a sensitive CO_2_ detector. This system allows nearly instantaneous determination of total VOC production by utilizing a platinum catalyst to completely and quantitatively oxidize headspace VOCs to CO_2_ in coordination with a CO_2_ detector. Measurement of respiratory CO_2_ by bypassing the catalyst allowed the total VOC content to be determined from the difference in the two signals. To the best of our knowledge, this is the first instance of a platinum catalyst and CO_2_ detector being used to quantify the total VOCs produced by a complex bioreactor system. Continuous recording of these CO_2_ data provided a record of respiration and total VOC production throughout the experiments. Proton transfer reaction-mass spectrometry (PTR-MS) was used to identify and quantify major VOCs. The sum of the individual compounds measured by PTR-MS can be compared to the total VOCs quantified by the platinum catalyst to identify potential differences in detection, identification and calibration. PTR-MS measurements accounted on average for 94 % of the total VOC carbon detected by the platinum catalyst and CO_2_ detector. In a model system, a VOC producing endophytic fungus *Nodulisporium* isolate TI-13 was grown in a solid state reactor utilizing the agricultural byproduct beet pulp as a substrate. Temporal changes in production of major volatile compounds (ethanol, methanol, acetaldehyde, terpenes, and terpenoids) were quantified by PTR-MS and compared to the total VOC measurements taken with the platinum catalyst and CO_2_ detector. This analytical system provided fast, consistent data for evaluating VOC production in the nonhomogeneous solid state reactor system.

**Electronic supplementary material:**

The online version of this article (doi:10.1186/s13568-016-0264-2) contains supplementary material, which is available to authorized users.

## Introduction

Fungi and bacteria produce hundreds of volatile organic compounds (VOCs) with industrial applications including biofuels, insecticides, quorum sensing and biocontrol, flavor and aroma compounds, antibacterials and antifungals (Hung et al. 2015; Kai et al. 2009; Morath et al. 2012; Strobel 2014). Bioprospecting has identified many new microorganisms that produce valuable VOCs, and the types and amounts of these compounds often change with substrate, culturing conditions and growth phase (Bunge et al. 2008; Kai et al. 2009; Morath et al. 2012; Strobel 2014). Higher yields of most microbial VOCs must be achieved to make these bioprocesses industrially viable, but fast and accurate analytical methods to determine the type and amount of VOCs produced are lacking (Morath et al. 2012). Efficient ways to screen new and genetically modified strains, and changes in culture conditions, are required to rapidly identify improvement to VOC yields (Morath et al. 2012).

Solid phase microextraction (SPME) coupled with gas chromatography–mass spectrometry (GC–MS) has been used to identify compounds produced by microbial fermentation (Mallette et al. 2012; Morath et al. 2012). With little sample preparation, SPME GC–MS readily identifies VOCs using compound libraries, but does a poor job of quantifying the amounts of compounds produced (Luchner et al. 2012; Mallette et al. 2012; Morath et al. 2012) and is not well suited to screen for increased yields of VOCs. Traditional extraction techniques to concentrate VOCs can be time intensive, and large organic solvent volumes may lead to poor resolution of volatile compounds (Kai et al. 2009; Morath et al. 2012). Further, extraction techniques may identify fewer VOCs than SPME GC–MS (Kai et al. 2009; Morath et al. 2012). Another analytical method, proton nuclear magnetic resonance can quantify VOCs quickly, but has poor sensitivity, cannot determine carbon length and some oxygenated VOCs cannot be measured because signals are confounded with sugar peaks (Mallette et al. 2014).

Proton transfer reaction-mass spectrometry (PTR-MS) is an effective analytical technique for measuring VOCs in the headspace of both liquid and nonhomogeneous solid state microbial reactor systems (Bunge et al. 2008; Ezra et al. 2004b; Luchner et al. 2012; Mallette et al. 2012). With PTR-MS, many VOCs can be quantified down to the 10–100 parts per trillion range with reliable results (Biasioli et al. 2011; de Gouw et al. 2003; Lindinger et al. 1998). PTR-MS provides data quickly including a likely compound identification, without the extensive preparatory laboratory work required for extractions (Ammann et al. 2004; Luchner et al. 2012). With real-time gas phase measurement capability, the PTR-MS readily ties VOC data to experimental conditions and temporal changes, and can non-invasively utilize reactor off-gas for measurements (Luchner et al. 2012; Romano et al. 2015).

Previously, PTR-MS has been used to explore microbial systems, but estimates of error in VOC quantification can be between 15 and 40 % depending on system conditions (Bunge et al. 2008; Ezra et al. 2004b; Luchner et al. 2012; Mallette et al. 2012; Romano et al. 2015; Schmidberger et al. 2014; Singer et al. 2009). Quantification of total VOC production with PTR-MS can be improved with calibration, but significant uncertainties may remain due to compounds that are not readily detected by PTR-MS (e.g. alkanes) and complex mixtures where multiple compounds with different sensitivity (calibration) factors contribute to the same mass to charge ratio.

Alternatively, the platinum catalyst and sensitive CO_2_ detector provide a robust VOC quantification system (Baasandorj et al. 2015; Veres et al. 2010) that confidently provides the total amount of gas phase organic carbon in complex mixtures of VOCs produced by microbial systems. The platinum catalyst oxidizes VOCs completely to CO_2_, which allows a sensitive CO_2_ detector to accurately quantify the total VOC production in real-time, as well as record respiratory CO_2_. This accurate VOC total can be used to quantify headspace carbon for carbon balances and as a real-time process monitoring tool. Additionally, the total VOC measurements can be compared to less certain total quantifications performed with other instruments, such as PTR-MS, to identify discrepancies in the system. To the best of our knowledge, this is the first time the combination of a platinum catalyst and CO_2_ detector has been used to quantify total headspace VOC production from a complex bioreactor system.

This paper demonstrates total VOC quantification in a non-homogenous solid state reactor system, where both growth phase and VOC data can be cumbersome to collect (Desgranges et al. 1991; Krishna 2005). The system described here provides a virtually instantaneous method for quantifying total VOC production.

## Materials and methods

### Experimental setup

**Fig. 1:**
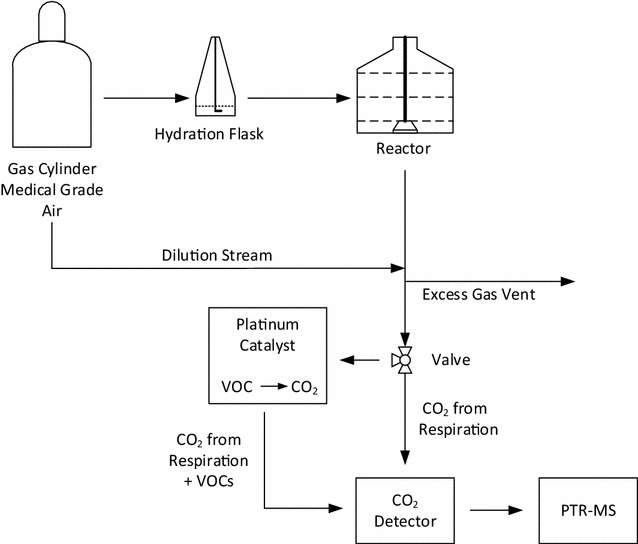
Experimental setup for monitoring VOC production from a fungal solid state reactor. Hydrated air was sparged through the bottom of the reactor. Reactor off-gas was sampled from the top of the reactor, diluted and sent to a CO_2_ detector to measure respiratory CO_2_ and then to the PTR-MS for quantification of major VOC species. Alternatively the off-gas was sent to a platinum catalyst where VOCs were oxidized to CO_2_ and measured by the CO_2_ detector as the sum of the VOCs produced and respiratory CO_2_

Figure 1 provides a schematic of the experimental setup. The platinum catalyst, CO_2_ detector and PTR-MS sampled approximately 200 mL/min of the diluted reactor off-gas stream. The flow path was alternated every 3 h using an automated three-way valve to flow either directly to the CO_2_ detector to measure respiratory CO_2_ only or through the VOC oxidizing platinum catalyst and then to the CO_2_ detector, measuring CO_2_ from both VOCs and fungal respiration. The PTR-MS sampled both the gas steams that entered and bypassed the platinum catalyst once per day. Gas flow that passed through the catalyst was measured to verify complete removal of VOCs from the gas steam and as a background with which to compare the bypass flow stream PTR-MS measurements. Three biological replicate experiments were performed to assess the reproducibility of the system.

### Solid state fungal reactor system

Solid state reactors constructed from a 2-L, air tight, borosilicate glass container with three stainless steel mesh shelves were inoculated with 250 mg of biomass as determined by a correlation with protein concentration using a modified Bradford method (Bradford 1976). As a substrate, 50 g of beet pulp were autoclaved for 20 min with 250 mL of water and allowed to cool. The reactor, hydration flask and all tubing were autoclaved at 121 °C for 20 min before use. Fungal biomass was added to the beet pulp, mixed by inversion and evenly distributed to the three shelves of the reactor. Biological triplicate experiments were run for approximately 5 days each until total VOC production approached zero.

The solid state reactor (see Fig. 1) was continuously sparged with 100 mL/min of hydrated medical grade compressed air. The air was delivered via a stainless steel tube (0.5 cm I.D.) running to the bottom center of the reactor. Inlet air pressure forced reactor gas out of the top of the reactor. Reactor off-gas was then diluted with 900 mL/min of dry medical grade air to keep water from condensing in the system and to keep concentrations within the linear range of the CO_2_ detector and PTR-MS. A portion of the diluted flow stream, ~200 mL/min, was then pulled through the CO_2_ detector and PTR-MS with the PTR-MS diaphragm pump.

### Microorganism

*Nodulisporium* isolate TI-13 was discovered as an endophyte of *Cassia fistula* in the highlands of Thailand using established collection and isolation methods as described previously (Ezra et al. 2004a). The TI-13 ITS1-5.8 S-ITS2 ribosomal gene sequence is available in GenBank as KJ558391, and the filamentous fungus is stored as sample NRRL 50502 in the Agriculture Research Service Culture Collection at the US Department of Agriculture (Nigg et al. 2014). The fungus was characterized as having the perfect stage of *Hypoxylon* sp. and as *Nodulisporium* sp. based on ITS1-5.8 S-ITS2 ribosomal gene sequence information (Nigg et al. 2014).

### Growth conditions

Inoculum cultures were generated by growing the fungus on potato dextrose agar until hyphae covered ≥60 % of the plate surface. A 20 % glycerol solution (10 mL) was added to each plate, scraped with a sterile glass rod and the suspended fungal biomass solution was collected. The solution was mixed thoroughly, added to Microbank microbead vials, and stored at −80 °C until use.

The composition of the inoculum medium was 60 g/L glucose and 0.5 g/L yeast extract. Two hundred and fifty millilitre of sterile filtered (0.22 µm) medium was added to 500 mL sterile baffled flasks. Three microbeads were added to each inoculum culture and grown for 9 days at 160 rpm and 30 °C. All inoculum flasks were covered with sterile Kimguard (Kimberly-Clark; Roswell, GA, USA) to allow gas exchange, but prevent microbial contamination.

### CO_2_ detector calibration and verification

An LI-840 CO_2_/H_2_O non-dispersive infrared gas analyzer (Li-cor Biosciences; Lincoln, NE, USA), quantified CO_2_ in the reactor off-gas continuously without impacting it. This optical technique is non-destructive and allowed the PTR-MS to be placed in series after the CO_2_ detector. The CO_2_ detector was calibrated using a certified CO_2_ free gas standard to set the zero point (1 % methane, balance nitrogen Scotty Analyzed Gases, Plumsteadville, PA, USA) and another gas standard for the span (a gas standard of 1010 ppm CO_2_, balance nitrogen, Scotty Analyzed Gases, Plumsteadville, PA, USA). Linearity of the CO_2_ detector was assessed by diluting the gas standard (1010 ppm CO_2_, balance nitrogen) with medical grade compressed air using mass flow controllers to produce final CO_2_ concentrations of 50.5, 101, 505 and 1010 ppm. The CO_2_ detector response agreed with the prepared gas standard to within 3.3 % for the four values measured, with values creating a line with a slope of 1.0006 and an R^2^ value of 0.9998 when plotting measured concentration versus expected concentration (data not shown).

### Platinum catalyst VOC conversion verification

Complete oxidation of VOCs to CO_2_ by a heated platinum catalyst (Shimadzu, Kyoto, Japan, High Sensitivity Catalyst 630-00996 maintained at 400 °C) was confirmed by diluting a gas standard (0.1 % propane, balance air, Cal Gas Direct, Huntington Beach, CA, USA) and medical grade compressed air with mass flow controllers to produce final propane concentrations in the range of 10–100 ppm, which spanned the total VOC concentration presented to the catalyst in the diluted sample flow. Based on the tenfold dilution of the bioreactor effluent and accounting for the three carbons in propane, this experiment produced a test matrix that was equivalent to total bioreactor VOC concentration of 300–3000 ppm C. Overall, the platinum catalyst and CO_2_ detector measurement agreed with the gas standard for conversion of propane to CO_2_ by the platinum catalyst to within 5 % for the four measured values, and created a line with a slope of 0.95 and an R^2^ value of 0.9991 when measured concentration was plotted against expected concentration. The catalyst exhibited near complete conversion efficiency for propane concentrations up to 50 ppm of propane, which decreased to 95 % at the highest, 100 ppm test point. This experiment established the effective maximum working concentration of the employed catalyst system at 1500 ppm C after adjusting for dilution. Catalyst systems employing higher temperatures and greater amounts of catalyst could be used to achieve higher maximum working concentrations. Similar catalyst systems have been used previously to confirm concentrations of calibration gases with high accuracy (Baasandorj et al. 2015; Veres et al. 2010).

### Bioreactor measurements

The reactor off-gas flow path was alternated with a valve on a 3 h timer to run either directly to the CO_2_ detector or through the platinum catalyst to oxidize VOCs to CO_2_ before the CO_2_ detector. By alternating the air stream either around or through the platinum catalyst every 3 h, a near-continuous record of respiratory CO_2_ and total gas phase carbon (CO_2_ and VOC) production was created for each experiment. The CO_2_ detector measurement was recorded once per second during the experiments. Background CO_2_ and VOCs measurements for each tank of medical grade compressed air were subtracted from these values.

### Proton transfer reaction-mass spectrometry (PTR-MS)

PTR-MS was used to provide compositional information about the VOCs in the reactor off-gas. PTR-MS uses H_3_O^+^ ions to protonate molecules (e.g. VOCs) with proton affinities greater than water (Lindinger et al. 1998). The singly charged ions are then typically detected as protonated molecules (ions with a mass-to-charge ratio, m/z, equal to the molecular weight plus 1 for the proton) by a quadrupole mass spectrometer (Lindinger et al. 1998). Identity is assigned based on the mass of the ion and prior knowledge of the likely products of a specific reactor system (Luchner et al. 2012). Constituents of air like O_2_, N_2_ and CO_2_ have proton affinities lower than water so are not protonated and do not interfere with measurements. Alkanes also have proton affinities lower than water, so PTR-MS does not efficiently detect alkanes (Lindinger et al. 1998).

Initial experiments with this solid state system showed the major VOC products were ethanol, methanol, acetaldehyde and monoterpenes, so care was taken to calibrate the PTR-MS for these compounds. Quantification was performed using sensitivity factors derived from calibration experiments by dynamically diluting a multicomponent standard containing methanol, acetaldehyde and terpenes (Apel-Riemer Environmental Inc., Broomfield, CO, USA). Ethanol calibration was performed separately using a permeation tube at a variety of humidities as described below. Sensitivity factors of 4.1, 17.4, 18.2 and 2.2 ncps (normalized counts per second)/ppbv for ethanol (m47, m65, m93), methanol (m33, m51), acetaldehyde (m45) and terpenes and terpenoids (m137) were used, respectively. The off-gas of the abiotic control and fungal cultures were both analyzed by the PTR-MS. The total VOCs measured in the abiotic control were subtracted from the fungal VOC production measurements.

### Ethanol PTR-MS calibration

Ethanol calibrations were performed using a calibrated permeation tube (KIN-TEK; La Marque, TX, USA) at a variety of humidity values approaching dry air as the experiments were performed with 90 % dry air. Compressed air (19.5 mL/min) was sparged through a temperature-controlled oven (50 °C) holding a calibrated ethanol permeation tube (emission rate 209 ng/min at 50 °C). Mass flow controllers were used to control medical grade compressed air flowing through the permeation tube and a second dilution stream, introduced after the permeation tube, to produce concentrations of 101, 199, 484 and 924 ppb of ethanol based on the calibrated permeation tube emission rate. The concentration of ethanol produced with the permeation tube was checked using the platinum catalyst system as described previously (Baasandorj et al. 2015) resulting in a line with a slope of 1.1 and an R^2^ value of 0.997 (data not shown). The temperature of the permeation tube was then increased to 80 °C to produce ethanol concentrations comparable to those observed in the fungal experiment (1.2–6.2 ppm). The humidity of the air was varied by utilizing a hydration flask before the dilution. Humidity was monitored via the ratio of the H_3_O^+^(H_2_O) to H_3_O^+^ ion intensities with calibration experiments performed under conditions that closely matched the humidity in the fungal experiments. At low humidity, the sensitivity factor had a relatively constant value of approximately 4.0. Calibrations were repeated after the experiments and yielded a sensitivity factor of approximately 3.9.

## Results

**Fig. 2:**
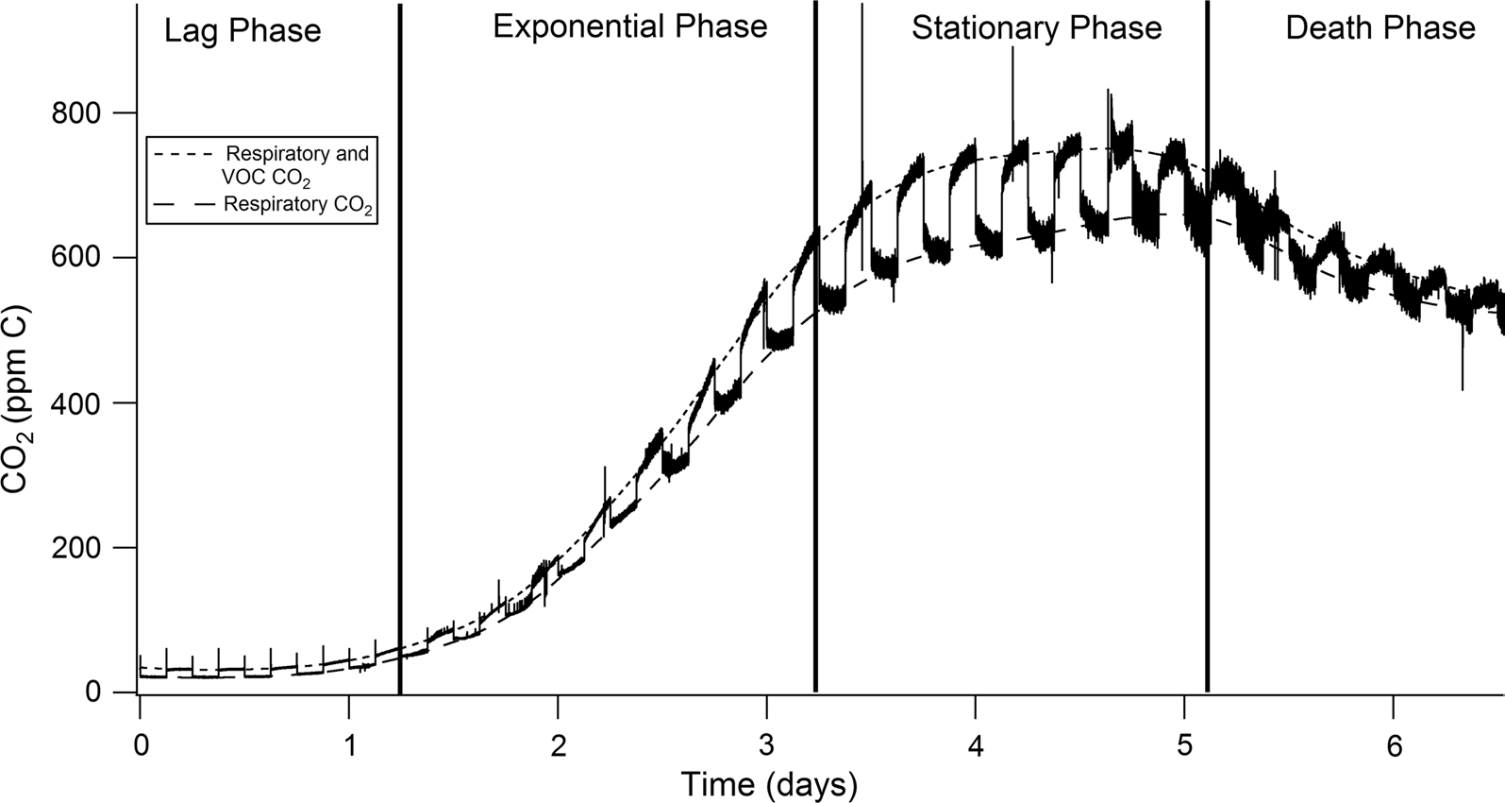
A representative CO_2_ profile of a fungal solid state reactor experiment after correcting for dilution. The *lower line* represents respiratory CO_2_ as indicated by the interpolated *dashed line* and the *upper line* is the sum of respiratory and oxidized VOC CO_2_ as represented by the interpolated *dotted line*. Values switch between respiratory CO_2_ and CO_2_ from both fungal metabolism and VOCs every 3 h. The graph is split into growth phases based on the shape of the respiratory CO_2_ curve

**Fig. 3:**
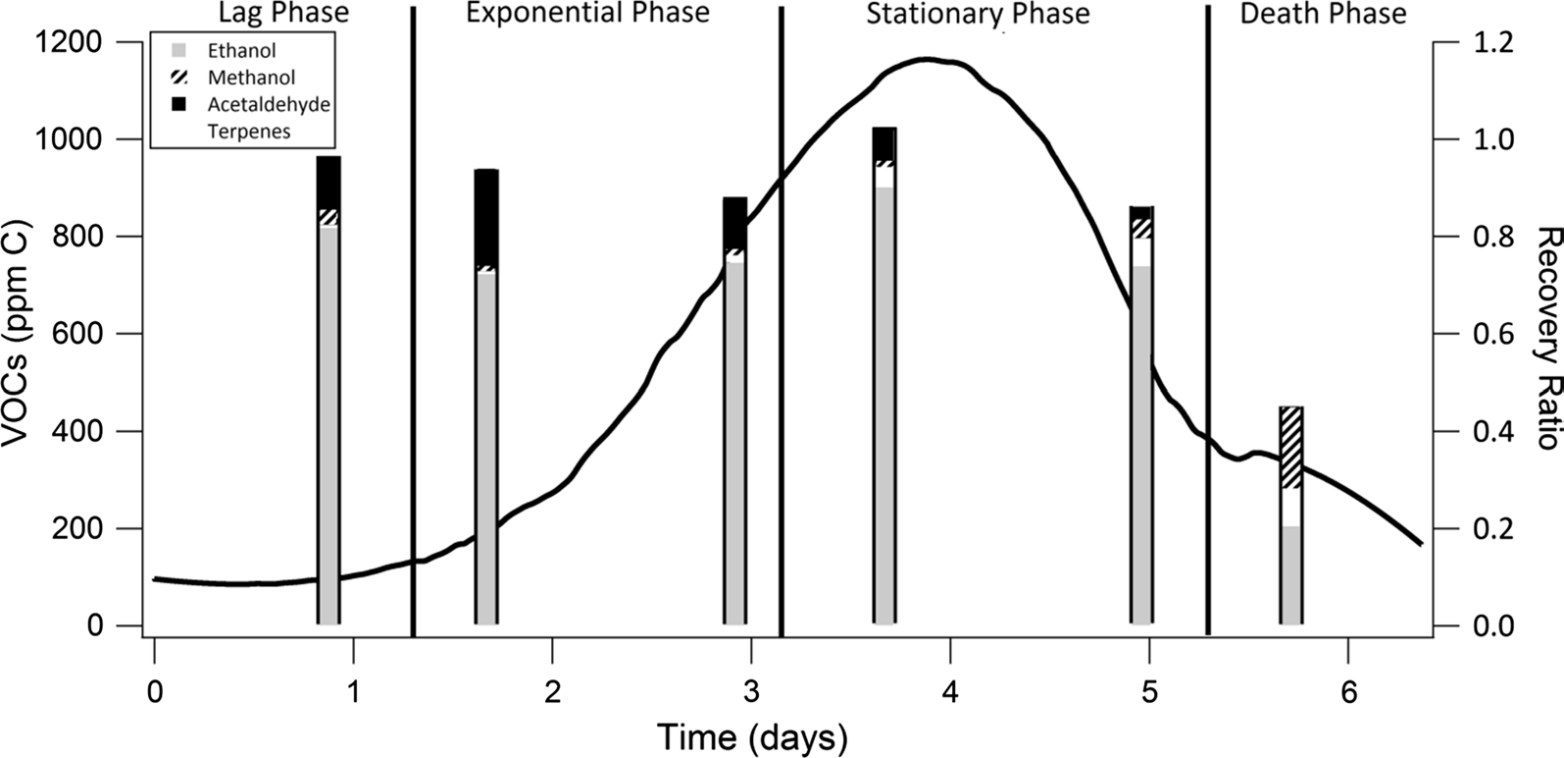
VOC production in the fungal reactor based on platinum catalyst VOC conversion to CO_2_ during experiment 3 corrected for dilution. Phase lines match the phases in Fig. 2. The *bars* represent PTR-MS measurements of ethanol (*grey*), terpenes (*white*), acetaldehyde (*black*) and methanol (*slash pattern*). The product areas are proportional to the relative percentage of carbon detected of each compound. The height of the bars represents the recovery ratio (shown on the *right axis*), which is the ratio of carbon detected by PTR-MS to carbon detected by the CO_2_ detector. A recovery ratio of one represents an equal amount of carbon detected

The record of respiratory CO_2_ and VOCs plus respiratory CO_2_ is shown as Fig. 2. The platinum catalyst oxidized these VOCs nearly completely to CO_2_ as demonstrated by the ±5 % conversion of propane to CO_2_ verification performed as described above. Additionally, no breakthrough was detected by the PTR-MS when the reactor off-gas was passed through the platinum catalyst, beyond decreasing system memory effects. The stair step shape of the CO_2_ data reflects the change of flow paths between going straight from the reactor to the CO_2_ detector, quantifying respiratory CO_2_ as the lower dashed line, and oxidation of VOCs to CO_2_ by the platinum catalyst before the CO_2_ detector, quantifying the sum of respiratory and VOC CO_2_ as the upper dotted line. The LOESS function of IGOR 6 (Wavemetrics; Portland, OR), a nonparametric regression method that smooths using locally weighted regression, was utilized to interpolate the lower line yielding a continuous respiratory CO_2_ record. The shape of the respiration lower line follows a typical growth curve and was used to estimate lag, exponential, stationary and death phases as shown in Fig. 2. Also, the lower line was integrated to determine the total amount of respiratory CO_2_ produced in parts per million carbon (ppm C) during each experiment and was then converted to the mass of carbon using the flow rate, temperature and pressure of the system. Similarly, the upper line, representing the sum of VOC production and respiratory CO_2_ in ppm C, was interpolated with the LOESS function to yield a continuous upper line. The lower line was subtracted from the upper line at each point in time to determine the VOC production in ppm C (Fig. 3). The area under the VOC production curve can be integrated to find the total amount of VOCs produced during the experiment in ppm C, which can then be converted to the mass of carbon using the flow rate, temperature and pressure of the system.

**Fig. 4:**
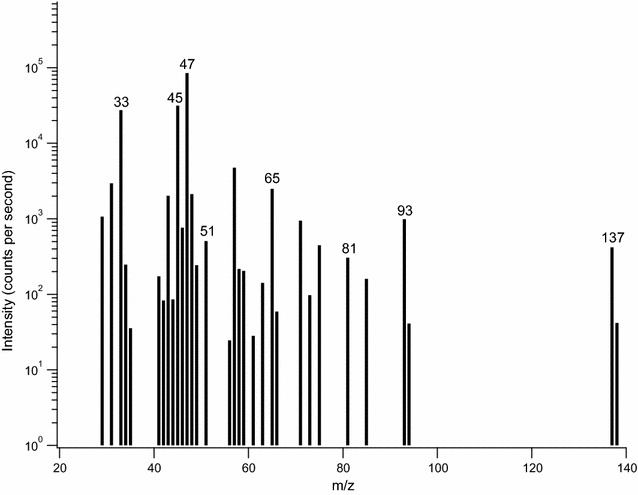
PTR-MS spectrum for experiment 3 (119 h) where background VOCs, reactor off-gas air sampled through the catalyst, have been subtracted from the reactor off-gas spectrum. The figure uses a logarithmic scale of intensity to aid in illustrating both major ions and ions of interest. Ions used for quantification of ethanol (47, 65, 93), methanol (33, 51), acetaldehyde (45) and terpenes and terpenoids (81, 137) are marked accordingly

Figure 3 shows the total VOCs quantified by the platinum catalyst and CO_2_ detector, which were extracted from Fig. 2 as described above. The sections of the bars displayed in Fig. 3 represent the relative carbon fractions of the major products as quantified by PTR-MS (as determined from spectra such as Fig. 4): ethanol, methanol, acetaldehyde, monoterpenes, and terpenoids (see Additional file 1: Table S1 for raw data). The predominant compound produced was ethanol in this and most of the samples. Total VOC production, as well as individual compound measurements, varied with growth phase of the fungus.

Additionally for these data, we can examine the VOC content quantified with PTR-MS for comparison to the total VOC production measured by the platinum catalyst and CO_2_ detector. Figure 4 shows a difference PTR-MS spectrum, where a background spectrum (obtained by measuring reactor off-gas passed through the catalyst) was subtracted from the off-gas measurements, from late stationary phase (119 h into experiment 3). Additional reactor off-gas VOC ions are present in the spectra, but they represented less than three percent of the ion intensity before death phase and were not quantified. Results show counts for ethanol (m/z 47, 65, 93) were highest for most spectra. Large amounts of methanol (m/z 33, 51) and acetaldehyde (m/z 45) ions are present in this spectrum. The terpene and terpenoid (m/z 81, 137) peaks and concentrations are smaller, despite accounting for more carbon per mole since they have ten carbons per molecule, compared to one or two carbon compounds like methanol and ethanol. Higher counts of two ions (m/z 57, 71), each of up to three percent of total ion intensity, and small but increased amounts of several other ions (less than one percent of total ion intensity each) were detected in death phase spectra (data not shown).

The compounds quantified by the PTR-MS (acetaldehyde, ethanol, methanol and terpenes) were multiplied by their carbon number and summed to determine the total concentration of VOCs in ppm C. This concentration was then divided by the total VOC production in ppm C determined by the platinum catalyst and CO_2_ detector to calculate recovery as shown by the height of the bars compared to one on the right axis in Fig. 3. Ideally, recovery ratios would be 1.0 if the PTR-MS detected all carbon containing compounds in the headspace. The average recovery ratio observed here was 0.945 ± 0.047 (average and standard error; see Additional file 1: Table S1) indicating that any unidentified VOC products constitute only a small fraction of the total VOC production. Recovery ratios were furthest from 1.0 at lag and death phases. Total VOC production was also lowest during lag and death phases, so small inaccuracies in CO_2_ concentration differences between bypass and catalyst measurements likely contributed to the recovery ratio error. Additionally, multiple small intensity ion signals that were not quantified appeared in death phase, which is expected due to cellular degradation and changing metabolism (Hazelwood et al. 2008). Fungal production of small concentrations of alkanes, which are undetectable by the PTR-MS, and small errors in calibration of the CO_2_ detector and PTR-MS may also contribute to errors in total quantification.

Two types of deviations from the anticipated response appeared in the CO_2_ detector signals later in the experiments. “Rolling” deviations in the CO_2_ signal were observed when switching between modes in which the signal exhibited some hysteresis and was slow to achieve a steady measurement immediately following the change in flow paths. This is most readily apparent starting in late-exponential phase (about 60 h) as observed in Fig. 2. The rolling characteristic was observed only in this experimental set and is likely due to pressure variations that occurred when switching between lines. Yegneswaran et al. (1990) observed similar patterns of CO_2_ concentrations and determined they were caused by small pressure changes in the bioreactor, but quickly returned to steady state. This type of deviation could be avoided by using two CO_2_ detectors. Additionally, some noise was observed in the infrared based CO_2_ measurements starting in late stationary phase and increased until the end of the experiments, but no cause was determined for this deviation. While this deviation persisted in later experiments, a definitive cause has not been determined. The combined momentary inaccuracies are estimated at less than 5 % of the signal, and do not significantly influence total VOC calculations for the majority of each experiment. During death phase, these perturbations were larger compared to the total signal and may contribute to less accurate death phase VOC recovery results.

### Respiratory CO_2_, VOC production and reproducibility

Three biological replicate experiments were performed to assess the reproducibility of the system. Total respiratory CO_2_, total organic carbon in the headspace (from VOCs) and substrate percentage of carbon converted to VOCs and CO_2_ were very reproducible for the three experiments. The total amount of respiratory CO_2_ produced was 0.73 ± 0.040 carbon g with three biological replicates (average and standard error) calculated by integrating the lower respiratory CO_2_ line in Fig. 2 as described above. Total VOC production was calculated in an analogous fashion by integrating data such as that shown in Fig. 3. Overall, 0.087 ± 0.0055 carbon g of total VOC were produced in each experiment. The average amount of beet pulp carbon (43 % carbon) (Stoppok and Buchholz 1985) converted to respiratory CO_2_ and VOCs was 3.8 ± 0.19 %. A biomass measurement assay was not utilized in these experiments. Whole experiment headspace selectivity was calculated as 0.12 ± 0.0080 by dividing the VOCs by the respiratory CO_2_ as measured by the CO_2_ detector throughout the experiment as described above.

## Discussion

The combination of PTR-MS, platinum catalyst and sensitive CO_2_ detector allowed for real-time VOC sampling with a quantitative determination of the compounds produced. The system described provides a robust measurement of the total VOC production as well as composition of major species produced by the non-homogenous solid state reactor system. To the best of our knowledge, this is the first time the total gas phase VOC content of a solid state reactor has been reported.

The platinum catalyst provides an accurate measurement of total headspace VOC production in complex microbial systems. Catalyst systems are effective at oxidizing nearly all the VOCs present in a sample, and are routinely used to purify air of VOCs for background measurements in PTR-MS (de Gouw and Warneke 2007) and to measure total organic carbon (Sugimura and Suzuki 1988). The platinum catalyst and CO_2_ detector agreed with the propane gas standard to within 5 % in our verification, and similar systems used to verify gas calibration standards agree to within 4–5 % of standards (Baasandorj et al. 2015; Veres et al. 2010). Catalyst systems are more accurate at measuring VOCs under many conditions than other detection systems currently available, such as PTR-MS and GC–MS (Ammann et al. 2004; Baasandorj et al. 2015; de Gouw et al. 2003; Kajos et al. 2015; Veres et al. 2010). An accurate total VOC measurement allows confident determination of the fraction one compound represents in a VOC mixture. Also, the platinum catalyst and CO_2_ detector are an affordable system to accurately quantify total headspace VOC carbon and respiratory CO_2_ for carbon balances. However, the platinum catalyst and CO_2_ detector do not provide any information on the identity of the VOCs present in a sample.

The platinum catalyst can also be used as an assessment tool to determine if the majority of VOCs produced are being detected, accurately identified and accurately quantified by the PTR-MS. A difference in the total VOC content determined by the platinum catalyst and CO_2_ detector from that provided by the PTR-MS can alert the user to check for issues such as the following: additional ions with significant carbon content may need to be quantified in the PTR-MS spectrum, some compounds (e.g. alkanes) are not being detected, sensitivity factors may be inaccurate, or identification of the VOC precursor responsible for part or all of an ion’s intensity may be incorrect. Ideally, all but one of these issues would be ruled out using prior system knowledge before using this assessment tool to identify discrepancies in PTR-MS VOC quantification. In this system, the PTR-MS total VOC content agreed on average to within 5.5 ± 4.7 % of that determined with the platinum catalyst and CO_2_ detector, which is considered to be excellent agreement. This level of agreement suggests that the PTR-MS quantification is providing an accurate measurement of VOC composition and that there are no significant errors in the calibration of the four major products, especially the detailed calibration of the major product, ethanol. The system described here is rapid and efficient at measuring the composition of VOCs with high confidence compared to other available measurement methods.

Identifying and quantifying headspace metabolites with PTR-MS throughout growth phases may aid in understanding metabolic processes (Bunge et al. 2008; Crespo et al. 2011; Luchner et al. 2012) and improve monitoring in production systems (Schmidberger et al. 2014). For example, acetaldehyde production appears to be an early marker of impending robust growth in some bacterial and fungal species and can be detected before increases in cell dry weight (Bunge et al. 2008) or respiratory CO_2_. Similarly, total VOC measurements can help verify robust growth, product formation and aid in understanding novel processes. This system can also determine headspace selectivity and productivity for total VOC production or individual compounds. For facilities without a PTR-MS, utilizing a platinum catalyst and CO_2_ detector in conjunction with SPME GC–MS could be employed. This would provide real-time respiratory CO_2_ and total VOC production rates, with SPME GC–MS used to identify specific compounds in complex VOC mixtures. While SPME GC–MS does not provide reliable quantification, it can be used in coordination with other assays to measure products (Mallette et al. 2012).

This total VOC quantification system can be applied in many different types of bioreactors. Bioremediation projects could utilize the detection system to quantify VOC removal in soil, water, or air samples. This system has applications in screening VOC producing organisms, VOC yield changes from varying process conditions, identifying appropriate target genes for genetic engineering and verifying increased VOC production in genetically engineered strains. The VOC measurement system could also be used for online process monitoring of bioreactors (Schmidberger et al. 2014), especially in solid state reactor systems where accurate and immediate online measurements are extremely difficult (Lui 2013). Similarly, many novel biological systems under study present challenges to traditional rapid monitoring methods, where complications with standard methods arise from a number of conditions such as complex carbon sources, fungi growing with pellet morphology and biofilms. These systems can benefit from utilizing the method described here to provide additional metabolic data in real-time.

## Additional file

10.1186/s13568-016-0264-2 Ethanol, methanol, acetaldehyde and terpene quantification data as measured by PTR-MS for each experiment and the abiotic control. Recovery ratios are also provided for each set of measurements.
