# Supplementary material for: Rapid total volatile organic carbon quantification from microbial fermentation using a platinum catalyst and proton transfer reaction-mass spectrometry
Source: AMB Express. 2016 Oct 5;6:90. doi: 10.1186/s13568-016-0264-2 (PMC5052237; doi:10.1186/s13568-016-0264-2)
Supplement: Supplementary file 1 — 10.1186/s13568-016-0264-2 Ethanol, methanol, acetaldehyde and terpene quantification data as measured by PTR-MS for each experiment and the abiotic control. Recovery ratios are also provided for each set of measurements. [file 13568_2016_264_MOESM1_ESM.docx]

**Supplemental Data**

Rapid total volatile organic carbon quantification from microbial fermentation using a platinum catalyst and proton transfer reaction-mass spectrometry

AMB Express

Heidi R. Schoen^1,2^, Brent M. Peyton^1,2^, W. Berk Knighton^3*^

1. Department of Chemical & Biological Engineering, 305 Cobleigh Hall, PO Box 173920, Montana State University, Bozeman MT 59717
2. Center for Biofilm Engineering, 366 Barnard Building, P.O. Box 173980, Montana State University, Bozeman MT 59717
3. Department of Chemistry and Biochemistry, 103 Chemistry and Biochemistry Building,
   PO Box 173400, Montana State University, Bozeman MT 59717

*To whom correspondence should be addressed:

Montana State University, 103 Chemistry and Biochemistry Building, PO Box 173400 Bozeman, MT 59717

Phone: (406) 994-5419, Fax: (406) 994-5407, Email: [bknighton@chemistry.montana.edu](mailto:bknighton@chemistry.montana.edu)

Table S1 Ethanol, methanol, acetaldehyde and terpene quantification data as measured by PTR-MS and VOC CO_2_ as measured by the CO_2_ detector. The recovery ratio is calculated as carbon in ppm detected by the PTR-MS divided by the carbon in ppm detected by the CO_2_ detector after oxidation by a platinum catalyst. The control in the bottom row is a sterile, uninoculated beet pulp reactor

| Experiment | Time (hours) | Phase | Ethanol (ppm C) | Methanol (ppm C) | Acetaldehyde (ppm C) | Terpenes (ppm C) | Total Carbon PTR-MS (ppm C) | VOC CO_2_ detector (ppm C) | Recovery Ratio |
| --- | --- | --- | --- | --- | --- | --- | --- | --- | --- |
| 1 | 18 | Lag | 9.73 | 0.308 | 1.59 | 0.00789 | 11.6 | 12.9 | 0.90 |
| 1 | 42 | Exponential | 33.4 | 0.206 | 5.24 | 0.209 | 39.1 | 39.1 | 1.00 |
| 1 | 66 | Exponential | 88.1 | 1.4 | 4.88 | 6.19 | 101 | 88.4 | 1.14 |
| 1 | 90 | Stationary | 49 | 3.7 | 1.4 | 7.23 | 61.3 | 54.8 | 1.12 |
| 1 | 114 | Death | 0.198 | 5.25 | 0 | 2.12 | 7.56 | 6.61 | 1.14 |
| 2 | 23 | Lag | 4.69 | 0.767 | 3.45 | 0.00399 | 5.8 | 8.08 | 0.72 |
| 2 | 49 | Exponential | 8.29 | 0.425 | 2.24 | 0.032 | 11 | 11.9 | 0.92 |
| 2 | 73 | Exponential | 32.1 | 0.33 | 4 | 0.731 | 37.1 | 39.2 | 0.95 |
| 2 | 97 | Stationary | 75.2 | 1.21 | 3.65 | 4.54 | 84.6 | 81.7 | 1.04 |
| 2 | 118 | Stationary | 64.4 | 2.34 | 1.82 | 5.21 | 73.8 | 68.4 | 1.08 |
| 3 | 21 | Lag | 4.18 | 0.153 | 0.582 | 0.00392 | 4.92 | 5.12 | 0.96 |
| 3 | 40 | Exponential | 6.57 | 0 | 1.88 | 0.0159 | 8.47 | 9.1 | 0.93 |
| 3 | 70 | Exponential | 31 | 0 | 4.5 | 0.825 | 36.3 | 41.6 | 0.87 |
| 3 | 88 | Stationary | 61.3 | 0.557 | 4.79 | 2.84 | 69.5 | 68.1 | 1.02 |
| 3 | 119 | Stationary | 49.7 | 2.52 | 1.98 | 3.77 | 57.9 | 67.3 | 0.86 |
| 3 | 137 | Death | 4.94 | 4 | 0.128 | 1.87 | 10.9 | 24.2 | 0.45 |
| 4 | 65.3 | Stationary | 83.6 | 1.9 | 3.91 | 7.59 | 97 | 89.6 | 1.08 |
| 4 | 98 | Death | 1.27 | 5.25 | 0 | 2.74 | 9.27 | 18.7 | 0.50 |
| 4 | 113 | Death | 0.13 | 5.28 | 0 | 1.06 | 6.36 | 5 | 1.27 |
| Control | 23 | Control | 0.0197 | 1.73 | 0.107 | 0 | 1.86 | 1.62 | 1.14 |
